# Identification of an Immune-Related Prognostic Risk Model in Glioblastoma

**DOI:** 10.3389/fgene.2022.926122

**Published:** 2022-06-17

**Authors:** Zhiying Lin, Rongsheng Wang, Cuilan Huang, Huiwei He, Chenghong Ouyang, Hainan Li, Zhiru Zhong, Jinghua Guo, Xiaohong Chen, Chunli Yang, Xiaogang Yang

**Affiliations:** Jiangxi Provincial People’s Hospital, The First Affiliated Hospital of Nanchang Medical College, Nanchang, China

**Keywords:** biomarker, infiltrated immune cell, glioblastoma, prognostic risk model, tumor immune microenvironment

## Abstract

**Background:** Glioblastoma (GBM) is the most common and malignant type of brain tumor. A large number of studies have shown that the immunotherapy of tumors is effective, but the immunotherapy effect of GBM is not poor. Thus, further research on the immune-related hub genes of GBM is extremely important.

**Methods:** The GBM highly correlated gene clusters were screened out by differential expression, mutation analysis, and weighted gene co-expression network analysis (WGCNA). Least absolute shrinkage and selection operator (LASSO) and proportional hazards model (COX) regressions were implemented to construct prognostic risk models. Survival, receiver operating characteristic (ROC) curve, and compound difference analyses of tumor mutation burden were used to further verify the prognostic risk model. Then, we predicted GBM patient responses to immunotherapy using the ESTIMATE algorithm, GSEA, and Tumor Immune Dysfunction and Exclusion (TIDE) algorithm.

**Results:** A total of 834 immune-related differentially expressed genes (DEGs) were identified. The five hub genes (STAT3, SEMA4F, GREM2, MDK, and SREBF1) were identified as the prognostic risk model (PRM) screened out by WGCNA and LASSO analysis of DEGs. In addition, the PRM has a significant positive correlation with immune cell infiltration of the tumor microenvironment (TME) and expression of critical immune checkpoints, indicating that the poor prognosis of patients is due to TIDE.

**Conclusion:** We constructed the PRM composed of five hub genes, which provided a new strategy for developing tumor immunotherapy.

## Introduction

Glioblastoma (GBM) accounts for 45.2% of primary malignant tumors in the central nervous system (Louis et al., 2016). GBM has remarkable communication ability with the tumor microenvironment (TME) and heterogeneity, which show a significant role in proliferation, invasion, and migration (Li G et al., 2017). Although significant progress has been made in the treatment of GBM, including surgery, radiotherapy, and chemotherapy, the prognosis of GBM is still unsatisfactory (Sathornsumetee et al., 2007; Onishi et al., 2011).

At present, immunotherapy for glioma is the most agreeable option, and a lot of related research is underway, such as programed cell death 1 ligand 1 (PDL-1) (Mathios et al., 2016), indoximod (IDO) (Lukas et al., 2019), and cytotoxic T lymphocyte antigen 4 (CTLA-4) (Fong et al., 2012). Increasing evidence shows that the effect of immunotherapy is not only related to tumor cells but also to the tumor microenvironment (TME) (Wu and Dai, 2017). Recent research has found that new immunoresponse therapies improve the prognosis of patients by enhancing the ability of the human immune system to recognize and attack tumor cells (Pitt et al., 2016; Gieryng et al., 2017).

In this study, we screened immune-related DEGs that are closely related to GBM and determined its prognostic value so as to investigate new GBM predictive models and potential biomarkers. Next, based on the TCGA database and CGGA database, a five-gene PRM that may be involved in immune infiltration was constructed. In addition, independent prognostic analysis, ROC curve and tumor mutation load analysis, and nomogram further verified the effect of the PRM in prognostic prediction. A robust immune-related PRM has been identified as an effective independent prognostic indicator for the subsequent personalized treatment of GBM.

## Materials and Methods

### Patients and Datasets

The gene expression matrix data, sample gene mutation data, and clinical information were downloaded from The Cancer Genome Atlas (TCGA) and Chinese Glioma Genome Atlas (CGGA). The CGGA dataset contained 374 GBM samples. The TCGA dataset contained 156 GBM samples and five normal samples.

### Screening of Immune-Related DEGs

The immune-related genes (IRGs) were obtained from the InnateDB database and Analysis Portal (ImmPort) database (Bhattacharya et al., 2014). A total of 6196 IRGs were used for further analysis. The immune-related DEGs were screened *via* the “pheatmap” and “limma” packages of the R language between normal and tumor tissues in GBM.

### Tumor Mutation Burden

The tumor mutation burden (TMB) score was calculated using “Maftools” packages of the R language. According to median data of the TMB score, we could divide GBM samples into high-and low-TMB groups.

### Weighted Gene Co-Expression Network Analysis

WGCNA was used to transform the association between genes and phenotypes into the association between some genomes and phenotypes *via* the R software package “WGCNA.”

### LASSO Analysis and Construction of a Prognostic Risk Model

The gene expression matrix of GBM patients in the TCGA database is defined as the training group, and that of GBM patients in the CGGA database is defined as the testing group. We carried out the regression analysis of the least absolute shrinkage and selection operator (LASSO). Then, we calculated the individualized risk score with the coefficient and constructed a prognostic risk model (PRM) to distinguish the high-risk group from the low-risk group. The PRM was established to evaluate the accuracy of the univariate prognostic model, and a multivariate prognostic model was built based on the area under the curve (AUC) of the receiver operating characteristic (ROC) curve using the “pROC” software package of R language.

Clinical characteristics and pathological features including gender, age, BRAF V600E, IDH status, Karnofsky performance status (KPS) scores, promoter methylation status of O6-methylguanine-DNA methyltransferase (MGMT), and original subtype were collected from the TCGA database. Multivariate proportional hazard model (COX) regression analysis proves the prognostic value of the risk score.

### Immunotherapy Response Prediction

The relative levels of abundance of the immune cell types were evaluated by the single sample gene set enrichment analysis (ssGSEA), which can quantify the scores of signature genes based on transcriptomic data (Hanzelmann et al., 2013). The CIBERSORT algorithm in the Tumor Immune Evaluation Resource (TIMER) online database is used to calculate the abundance of immune cells (Newman et al., 2015; Li T. et al., 2017). Furthermore, the Tumor Immune Dysfunction and Exclusion (TIDE) score was used to model the primary mechanisms of tumor immune evasion.

### Gene Set Enrichment Analysis

GSEA was used to analyze the biological function of a single gene. To analyze the main function of the different genes, the “clusterProfiler” package was used for GO and KEGG analyses.

### The Establishment and Evaluation of the Nomogram

The nomogram is used to integrate the related factors of tumor recurrence. The prediction ability of the model is further evaluated and quantified using the calibration curve of the nomogram.

## Results

**FIGURE 1:**
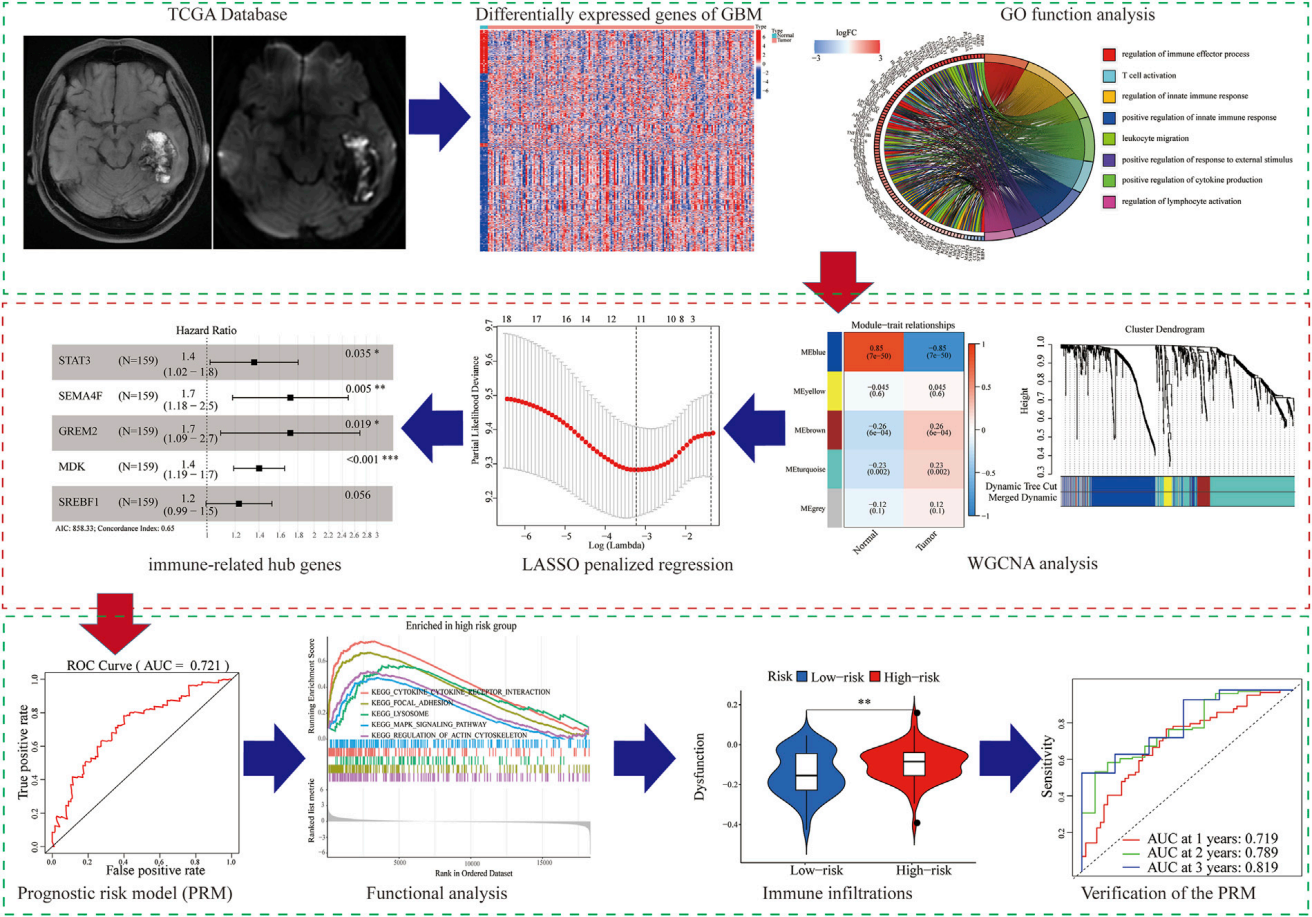
Flowchart of the workflow of the immune-related PRM.

In our study, we analyzed and verified a PRM based on differentially expression profiling of immune-related genes that may be used to aid prognostic analysis in patients with GBM. The PRM was associated with immune infiltration, immune checkpoint gene expression, and clinical characteristics. In summary, the risk model in our study can be used as a prognostic immune biomarker for GBM (Figure 1).

### Screening for Immune-Related DEGs and Functional Analysis

**FIGURE 2:**
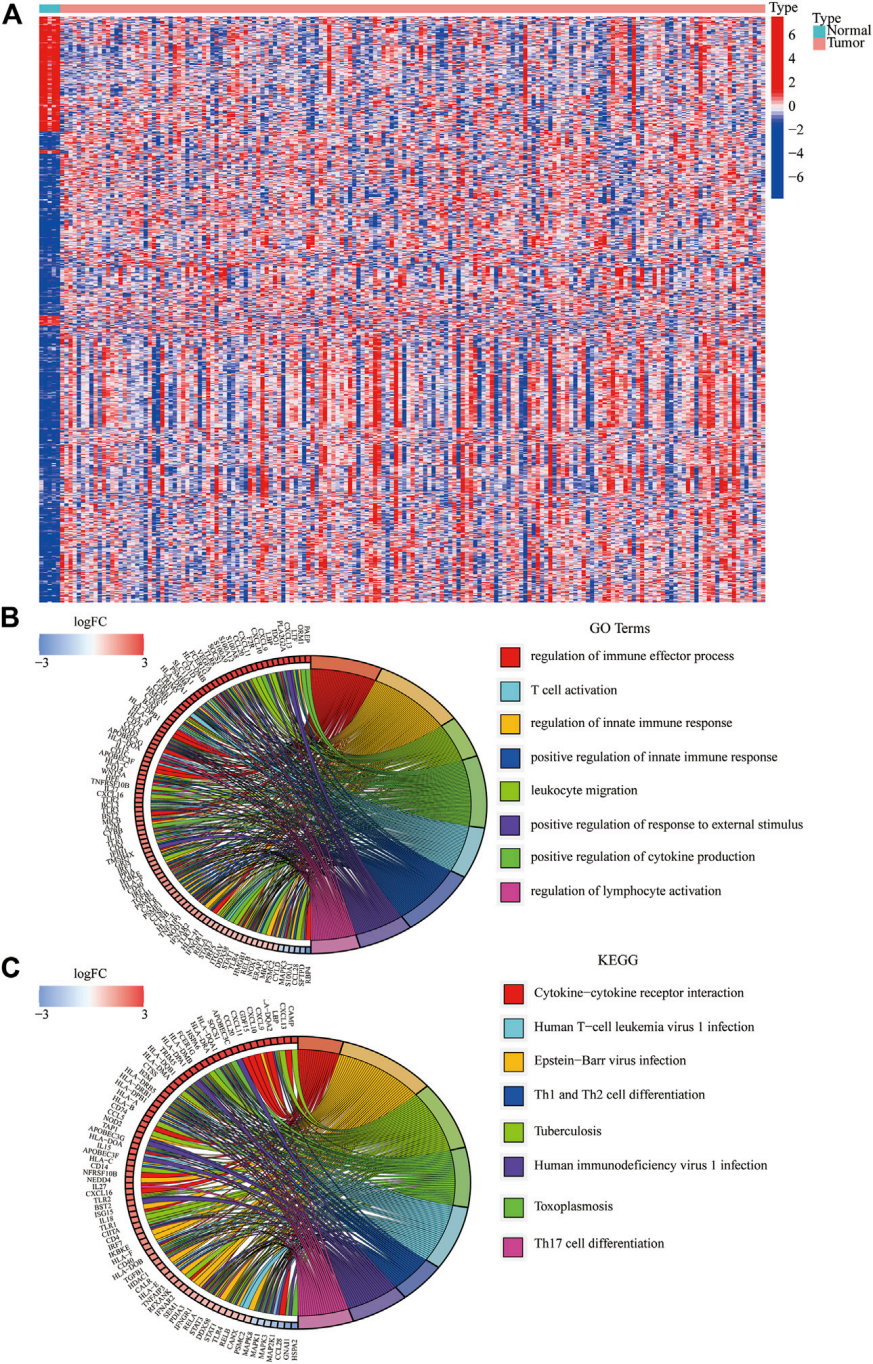
Immune-related DEGs in GBM from the TCGA database. **(A)** Heatmap visualizing the DEGs screened using the “limma” package. **(B,C)** Functional enrichment analysis of DEGs.

We identified 834 immune-DEGs from the TCGA database. The DEGs comprised 652 upregulated genes and 182 downregulated genes, using the criteria of |log_2_(FC)| > 1 (Figure 2A).

We annotated the functions of immune-related DEGs using GO functional analysis and KEGG enrichment analysis. The result of GO functional analysis for biological process analysis indicated that the DEGs are enriched in T-cell activation, regulation of the immune effector process, and regulation of innate immune response, (Figure 2B, *p* < 0.05). Furthermore, KEGG enrichment pathway analysis also demonstrated that the immune-related DEGs are mainly enriched in Th17 cell differentiation, Th1 and Th2 cell differentiation, and cytokine–cytokine receptor interaction (Figure 2C).

### WGCNA Analysis to Select the Co-Expression Modules and Hub Genes

**FIGURE 3:**
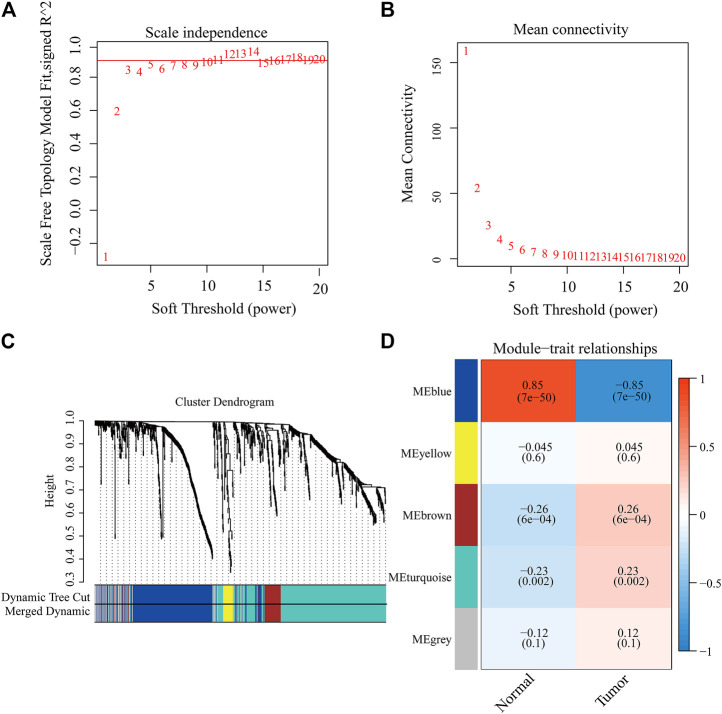
Identification of modules associated with the clinical traits of GBM in the WGCNA. **(A)** Analysis of the scale-free index for various soft-threshold powers (*β*). **(B)** Analysis of the mean connectivity for various soft-threshold powers. **(C)** Dendrogram of all differentially expressed genes clustered based on the measurement of dissimilarity (1-TOM). **(D)** Heatmap of the correlation between the module eigengenes and CDCP1 expression level of GBM. The color band shows the results obtained from the automatic single-block analysis.

We tried to use WGCNA to highlight the gene partial correlation. We used the expression matrix of GBM patients in the TCGA database to perform WGCNA analysis. Consequently, we built the adjacency matrix and constructed the topological overlap matrix (Figures 3A,B). Finally, three modules were identified based on average hierarchical clustering and dynamic tree clipping (Figure 3C). The MEblue, MEbrown, and MEturquoise modules were related to tumor development, which contained 289, 56, and 391 genes, respectively (Figure 3D). Interestingly, MEblue, which is the most statistically significantly different module, was also the most correlated module (correlation coefficient = 0.85, *p* < 0.001). The complex PPI network of the MEblue module consists of 95 nodes and 1,690 edges (Supplementary Figure S1).

### Construction of a Prognostic Model

**FIGURE 4:**
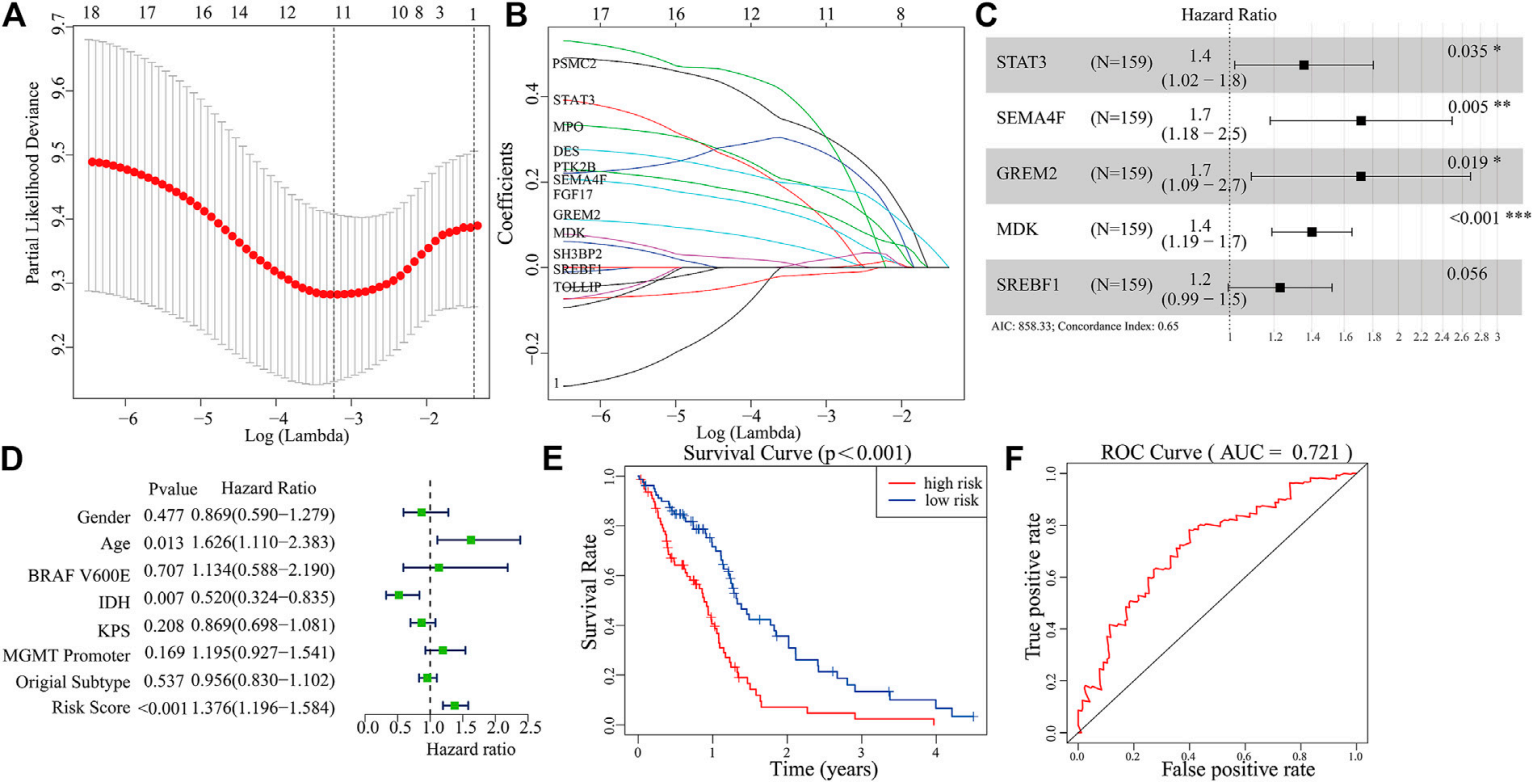
Construction of a prognostic model based on the 289 genes of the MEblue module in the training set. **(A,B)** Twelve survival-related genes by LASSO penalized regression. **(C)** Five potential prognostic genes *via* multiple Cox regression with LASSO penalty. **(D,E)** Prognostic classifier analysis of the patients in the internal testing set. **(F)** ROC curve of the potential prognostic genes.

A total of 289 genes of the MEblue module were selected to perform LASSO and COX regression. The TCGA cohort and CGGA cohort were defined as the training group and testing group, respectively. Furthermore, the 12 key genes (PSMC2, STAT3, MPO, DES, PTK2B, SEMA4F, FGF17, GREM2, MDK, SH3BP2, SREBF1, and TOLLIP) were constructed with LASSO regression, when the log value (lambda) was between -3 and -4 (Figures 4A,B). The Akaike information criterion (AIC) value is used for further analysis by multivariate COX regression with LASSO penalty (Table 1). Then, we screened out the core gene with the minimum AIC value and constructed a prognostic risk model comprising core genes. Using this method, we obtained five potential prognostic genes as hub genes, namely, STAT3, SEMA4F, GREM2, MDK, and SREBF1 (Figure 4C). We established the PRM using the selected hub genes STAT3, SEMA4F, GREM2, MDK, and SREBF1. By excluding the influence of gender, age, BRAF V600E, IDH status, KPS score, methylation status of MGMT promoter, and original subtype on prognosis, the PRM is substantiated to be an independent prognostic risk factor for GBM patients. The result showed that the hazard ratio (HR) of the PRM was 1.41 (95% confidence interval, CI: 1.20–1.58) in the TCGA database (Figure 4D). Further analysis of hub genes showed that the survival time of high-risk patients was significantly less than that of the low-expression group (Figure 4E). As shown by the ROC curve of the PRM in Figure 3F, the AUC value was 0.72. We further verified the reliability of the PRM through CGGA database prognostic analysis and ROC curve analysis (Supplementary Figures S2A,B).

**TABLE 1:** Details of features selected by the multivariate Cox proportional hazard regression model with LASSO penalty.

Gene	COFF	HR	*p*
STAT3	0.305	1.357	0.035
SEMA4F	0.540	1.716	0.005
GREM2	0.539	1.715	0.019
MDK	0.338	1.402	<0.001
SREBF1	0.207	1.230	0.056

HR, hazard ratio

We further analyzed the correlation between the PRM and clinical features (gender, age, BRAF V600E, IDH, KPS, MGMT, and original subtype) and tumor mutation burden (TMB). The results showed that GBM patients in the high-risk group were older, MES-GBM accounted for a larger proportion, and PN-GBM was lower (Supplementary Figure S3A). Also, TMB of the low-expression group is lower (Supplementary Figure S3B).

### Biological Function of the Prognostic Risk Model

**FIGURE 5:**
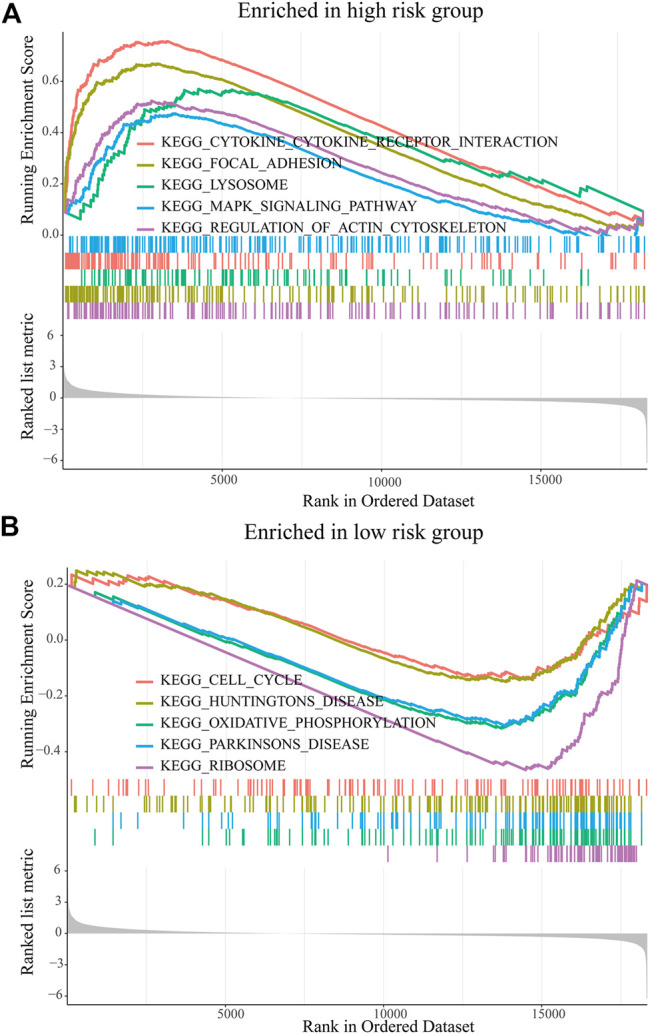
GSEA of KEGG pathway enrichment analysis of the prognostic risk model (PRM) in the TCGA database. **(A)** High-risk group. **(B)** Low-risk group.

The GSEA was used to predict the possible biological functions of the PRM in the TCGA dataset. The KEGG pathway enrichment analysis showed that high expression of the PRM was significantly correlated with focal adhesion, MAPK signaling pathway, and regulation of actin cytoskeleton (Figure 5A) and the low expression of the PRM was significantly correlated with cell cycle and oxidative phosphorylation (Figure 5B). The GO enrichment analysis indicated that the PRM was correlated with the cellular response to hormone stimulus, peptide transport, and cytochrome complex (Supplementary Figures S4A,B).

### Risk Score Was Correlated With Genomic Aberration Features

**FIGURE 6:**
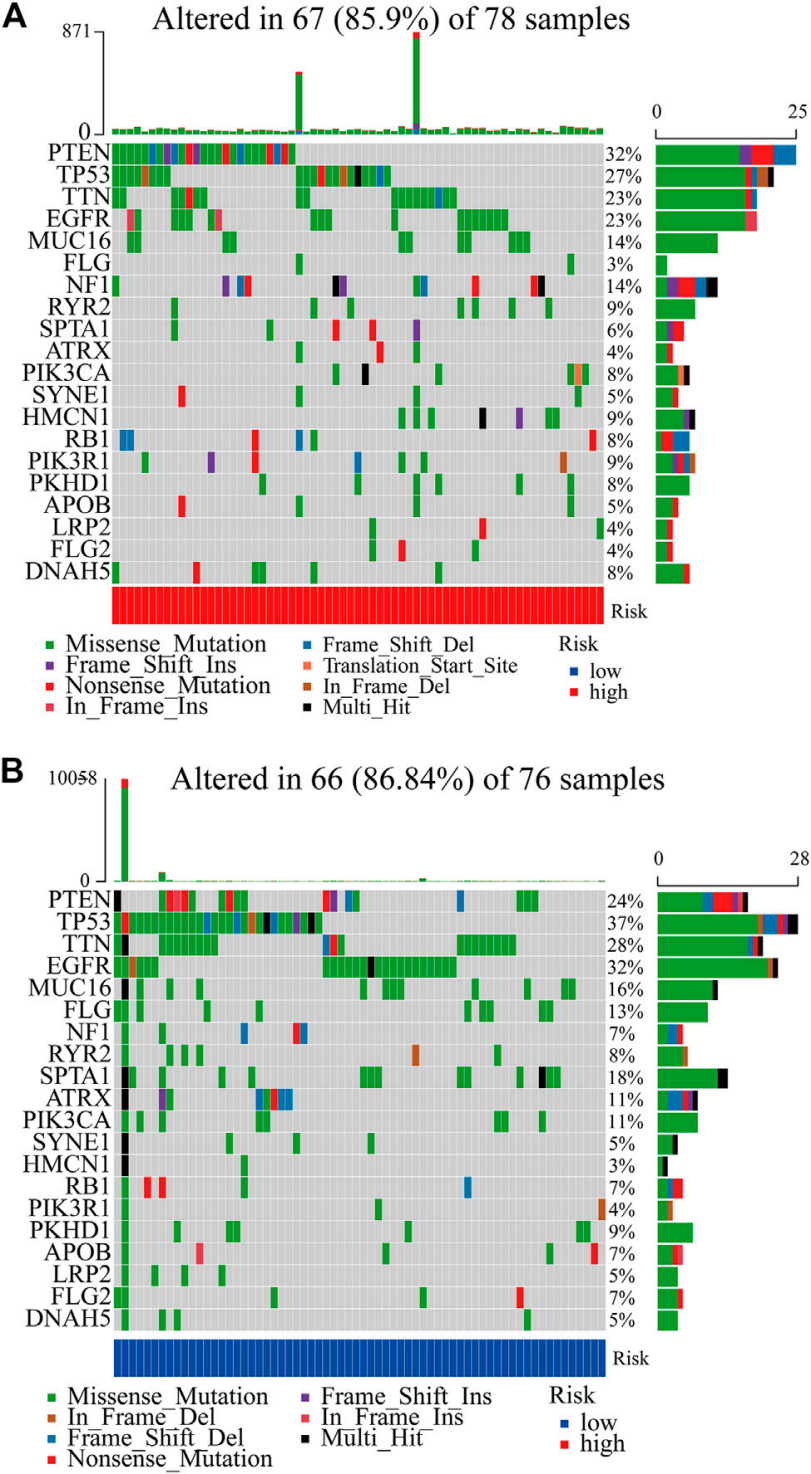
Heatmap of tumor mutation burden of the PRM in the TCGA database. **(A)** High-risk group. **(B)** Low-risk group.

In total, we used the “maftools” package to analyze the tumor mutation profiles of high PRM expression and low PRM expression. As shown in the waterfall plot, the tumor mutation burden was observed in 67 (85.90%) samples of the high-risk group and in 66 (86.84%) samples of the low-risk group. PTEN, TP53, TTN, EGFR, and MUC16 are the top five mutated genes in high-expression PRM group samples, and PTEN mutations are found in more than 30% of high-expression PRM group samples (Figure 6A). In the low-expression PRM group patients, TP53, EGFR, TTN, PTEN, and MUC16 are the top five mutant genes. The TP53 mutations are found in more than 35% of low-expression PRM group samples (Figure 6B).

### Immune Infiltration Landscape

**FIGURE 7:**
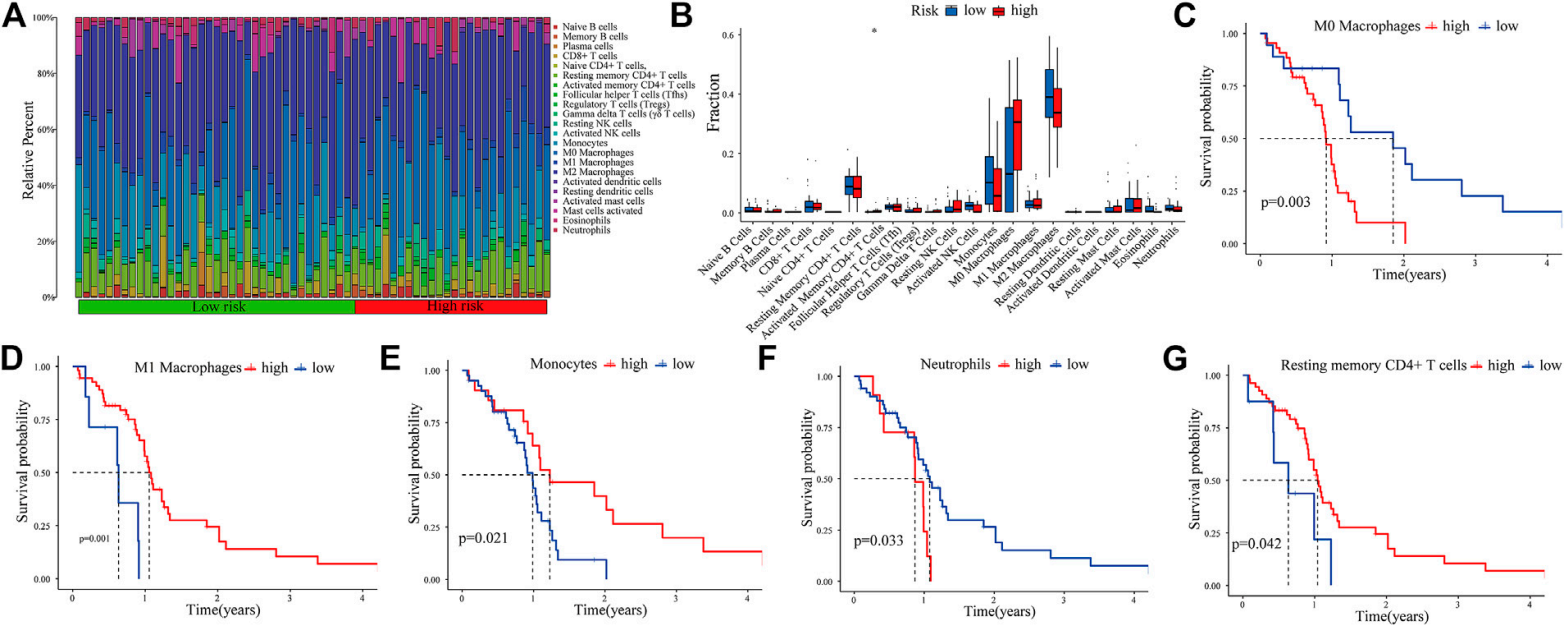
Immune infiltration in GBM samples as assessed in CGGA data. The proportions of tumor-infiltrating immune cells (TIICs) in 22 GBM patients from the CGGA database **(A)**. Correlation analysis between CDCP1 expression and various types of infiltrating immune cells **(B)**. Survival analysis of the TIIC subsets **(C–G)**.

The CIBERSORT algorithm was used to analyze the immune infiltration in GBM tissues. Figure 7A shows the proportions of immune cells in each GBM sample in different colors, and the lengths of the bars in the bar chart indicate the levels of the immune cell populations. Compared with the low-expression PRM group, we identified that the high-expression PRM had relatively high percentages of activated CD4^+^ memory T cells (Figure 7B). The results show that the difference in the tumor-infiltrating immune cell (TIIC) subgroup level among individuals partly reflects the prognosis. As shown in Figure 7, M0 macrophages and neutrophils were negatively correlated to overall survival (OS) in patients with glioma (Figures 7C,F). However, M1 macrophages, resting memory CD4^+^ T cells, and monocytes were positively related to OS (Figures 7D,E,G). The study suggests that the TIIC subgroup can provide the potential prognostic value for GBM treatment.

**FIGURE 8:**
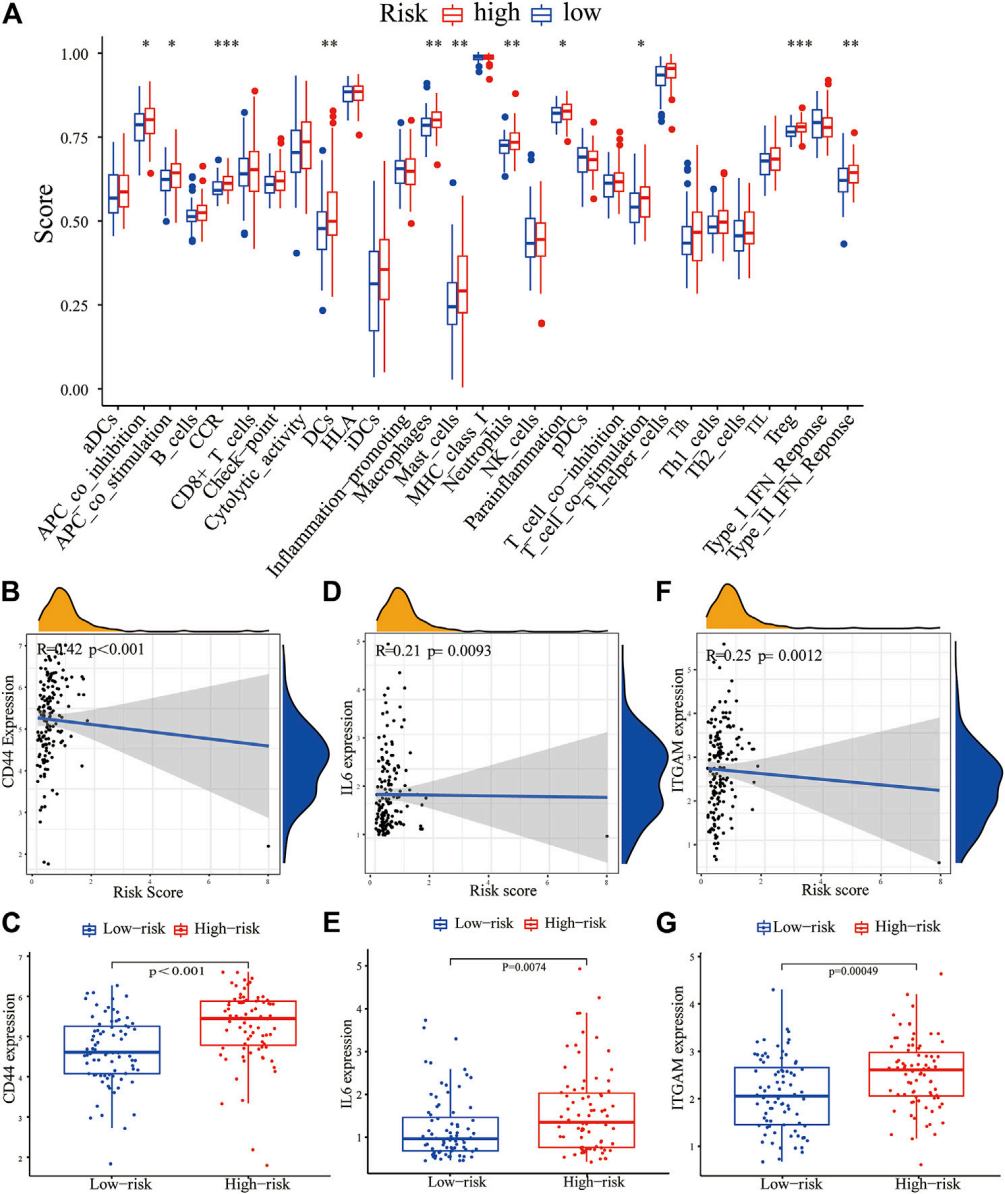
Correlation between the PRM and tumor immune microenvironment features. **(A)** Enrichment scores of 16 immune cells and 13 immune functions in the high-risk and low-risk groups of the PRM. The differential expression of most checkpoint genes, CD44 **(B,C)**, IL-6 **(D,E)**, and ITGAM **(F,G)** in the high-risk group and the low-risk group.

We further evaluated the correlation between the PRM and the characteristics of the tumor immune microenvironment through the “GSVA” package of R language. The result showed significant differences in immune cell infiltration and immune function, especially for regulatory T (Treg) cells and dendritic cells (DCs). Moreover, the higher CCR, parainflammation, and T-cell stimulation scores and type II IFN response scores were present in the high-expression PRM group rather than the low-expression PRM group (Figure 8A). We further verified the prognostic implications of immune cell infiltration and immune function by overall survival (OS) (Supplementary Figures 5A–W).

**FIGURE 9:**
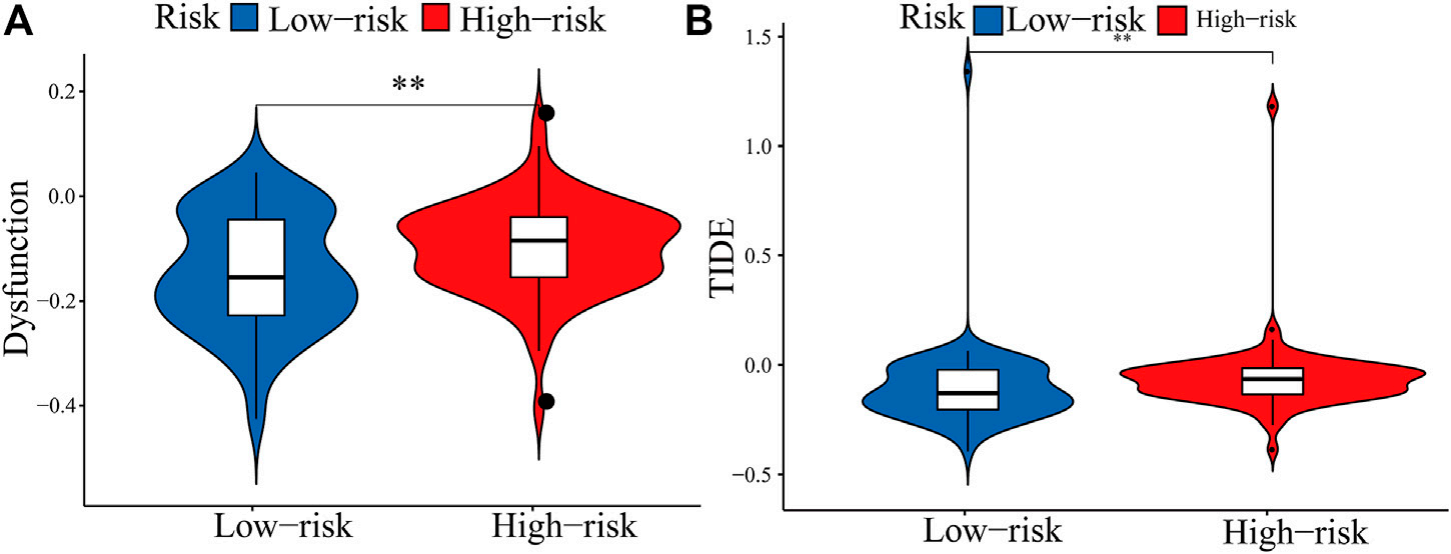
Boxplots showed the scores of immune infiltrations and functions among the PRM. **(A)** Dysfunction. **(B)** TIDE.

The expression of immune checkpoint genes, which play a key role in cellular immune regulation, in the PRM was further studied. It was found that compared with the low-risk group, the expression of most checkpoint genes (such as CD44, IL-6, and ITGAM) was upregulated in the high-risk group (Figures 7B–G). In conclusion, the consistency between PRM prognosis and TME characteristics suggests that this classification is reliable and reasonable. The dysfunction and TIDE scores were significantly higher for the high-risk group than for the low-risk group (Figures 9A,B).

### Establishment and Evaluation of Clinical Predictive Models

**FIGURE 10:**
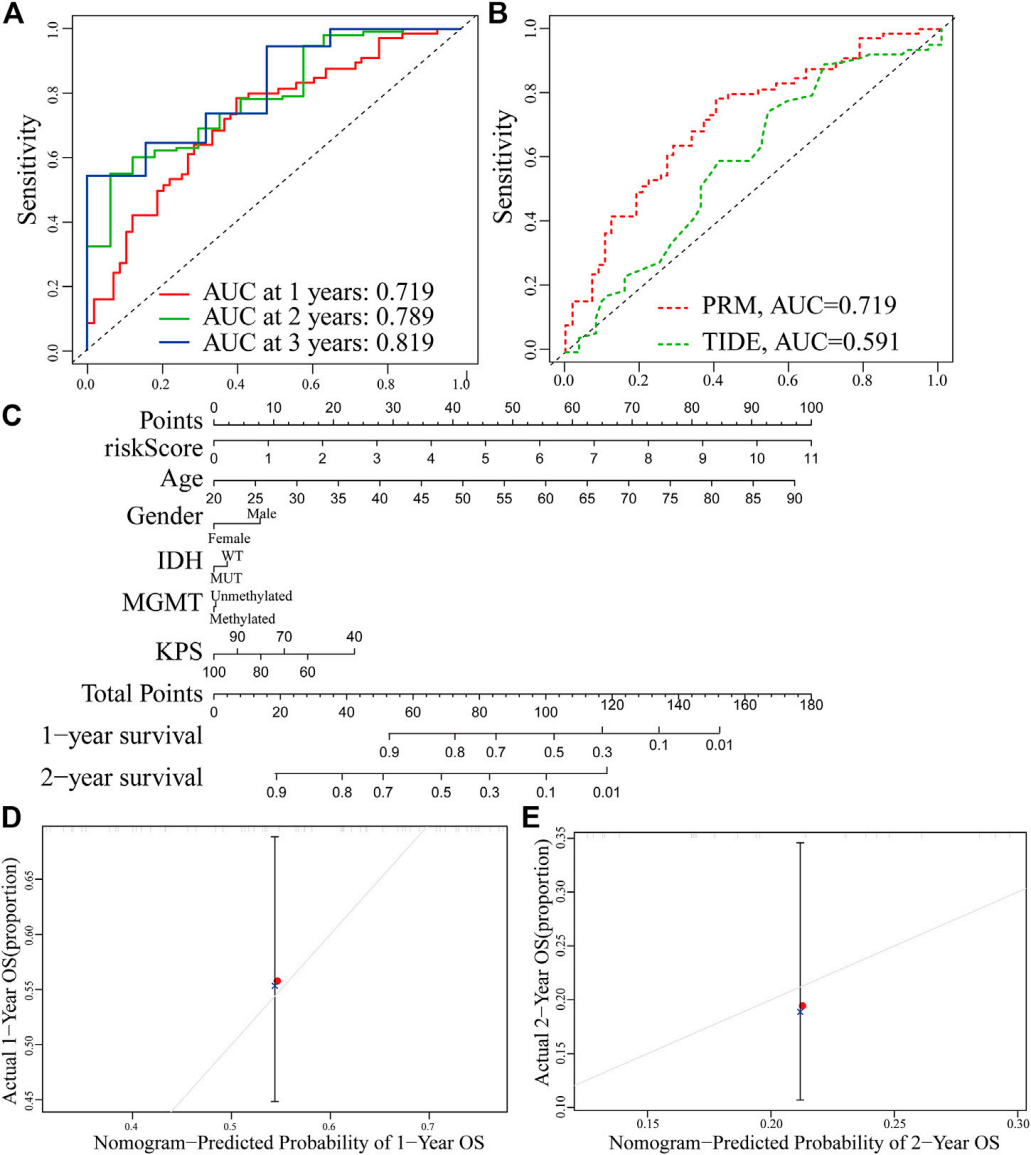
Establishment and verification of the prognostic risk model. **(A)** ROC curve of the PRM comprising STAT3, SEMA4F, GREM2, MDK, and SREBF1 expression at 1-year survival, 2-year survival, and 3-year survival. **(B)** Comparison between the traditional TIDE model and prognostic risk model. **(C)** Nomogram based on the PRM and clinicopathological factors. Calibration plot evaluating the predictive accuracy of the nomogram at 1-year survival **(D)** and 2-year survival **(E)**.

The receiver operating characteristic (ROC) curve showed that the AUC of the 1-, 2-and 3-year survival rate of PRM was greater than 0.7 and the AUC of the 3-year survival rate was 0.819, which indicated the superiority of our method (Figure 10A). The result shows that our PRM can accurately indicate the prognosis of GBM patients. The ROC curves were used to evaluate the predictive efficacy of the PRM and the TIDE. The AUC values for the PRM and TIDE were 0.719 and 0.591, respectively (Figure 10B).

The PRM and the clinical relevance and prognostic value of age, gender, IDH status, methylation status of MGMT promoter, and KPS scores were combined to construct a nomogram. Each factor in this nomogram is given a certain score (Figure 9C). The analysis of the nomogram and calibration curve proved that the PRM is reliable and accurate (Figures 10D,E). On the other hand, by comparing the factors in the nomograms, we found that the prognostic risk model had a high score, and this model played an important role.

## Discussion

Glioblastoma (GBM) is the most common malignant tumor in the central nervous system (CNS) (Davis, 2018), and there is no targeted therapy to ensure the maximum survival rate of glioma patients (Filbin and Suva, 2016; Louis et al., 2016). In the recent years, a large number of researchers used bioinformatics to analyze the data of thousands of expressed genes in the human genome through high-throughput sequencing and microarray analysis, which can be used to identify the immune-related gene characteristics existing in GBM and reveal its potential mechanism (Zhou et al., 2018).

As the basic unit of the immune system, cells are usually heterogeneous in the analyzed samples. The CIBERSORT algorithm was used to identify cell types so as to capture the background centered on cells and at the whole system level. Researchers have performed a lot of research to verify the effectiveness of the calculation method. The composition of immune cells in cancer tissues has been verified and successfully evaluated by flow cytometry and other methods (Cackowski et al., 2019). Infiltrating immune cells play an important role in promoting and/or regulating tumor progression and growth (Whiteside, 2008). These tumor immune cells produce various cytokines and chemokines, which are necessary for infiltrating immune cells to function in promoting inflammation or eliminating inflammation and have a great influence on the progress of glioma and the drug resistance of therapeutic intervention (Boussiotis and Charest, 2018). Our study and the existing literature report on the immune-related PRM of tumors all use R language to analyze the gene expression matrix of the public database, but we added LASSO regression, multivariate Cox regression analysis, and WGCNA analysis and further used nomograms to verify the model (Chen et al., 2020; Qian et al., 2021). The immune-related genes selected in this study are specific markers.

We finally screened five genes (STAT3, SEMA4F, GREM2, MDK, and SREBF1) by WGCNA and LASSO analysis of immune-related DEGs. The gene signal transducer and activator of transcription 3 (STAT3, Gene ID: 6774) is a transcription factor that is activated by various signal-induced phosphorylation. In the microenvironment of glioma and in the tumor microenvironment, the EGFR and the IL6 signaling pathway play important roles in activating STAT3 (Wang et al., 2013; Kim et al., 2016). STAT3 is abnormally activated in various immune cells, creating a microenvironment of immune escape (Wang et al., 2004; Melillo et al., 2010). The gene ssemaphorin 4F (SEMA4F, Gene ID: 10505) encodes a transmembrane class IV semaphorin family protein, which plays a role in neural development (Gabrovska et al., 2011; Shergalis et al., 2018), and the previous study found that SEMA3B was found to be a marker for poor survival in patients over 50 diagnosed with GBM (Rich et al., 2005). Gremlin-2 (GREM2, Gene ID: 64388) has been found to have the highest concentration in the brain but much lower in the kidney and lung (Church et al., 2017). It can inhibit the canceration and progression of endometrial cancer (Sun et al., 2020). Midkine (MDK, Gene ID: 4192) encodes a member of a small family of secreted growth factors that binds heparin and responds to retinoic acid (Guo et al., 2020). Sterol regulatory element binding transcription factor 1 (SREBF1, Gene ID: 6720) is essential for squamous cell carcinoma (SCC) viability and migration, and its overexpression is associated with poor survival in SCC patients (Li et al., 2021).

In our study, immune-related differential genes were screened out through differential expression, and then the PRM was constructed through bioinformatics. It was verified that the PRM was significantly positively correlated with immune cell infiltration and the expression of key immune checkpoints in the TME. These preliminary results provide a perspective for exploring the role of immune escape in GBM. However, this study has the following limitations. First of all, our research lacks further verification by *in vivo* experiments. Second, this research study is based on the public database, lacking the analysis of sequencing data in our institution to verify our research results.

In conclusion, we identified a five-gene prognostic risk model based on the differential expression profiling of immune-related genes that may be used to aid prognostic analysis in patients with GBM. The low-risk and high-risk groups of the PRM exhibit significant differences with respect to immune infiltration, TMB, and tumor immune evasion. The nomogram established and validated to the PRM is not only reliable but also showed that the accuracy of predicting survival in each patient was high. These findings provide novel insights into the design of immunotherapeutic strategies against GBM.

## Data Availability

Publicly available datasets were analyzed in this study. These data can be found here: https://cancergenome.nih.gov/ and http://www.cgga.org.cn/.
