# Supplementary figures and images for: Identification of an Immune-Related Prognostic Risk Model in Glioblastoma
Source: Front Genet. 2022 Jun 17;13:926122. doi: 10.3389/fgene.2022.926122 (PMC9247349; doi:10.3389/fgene.2022.926122)

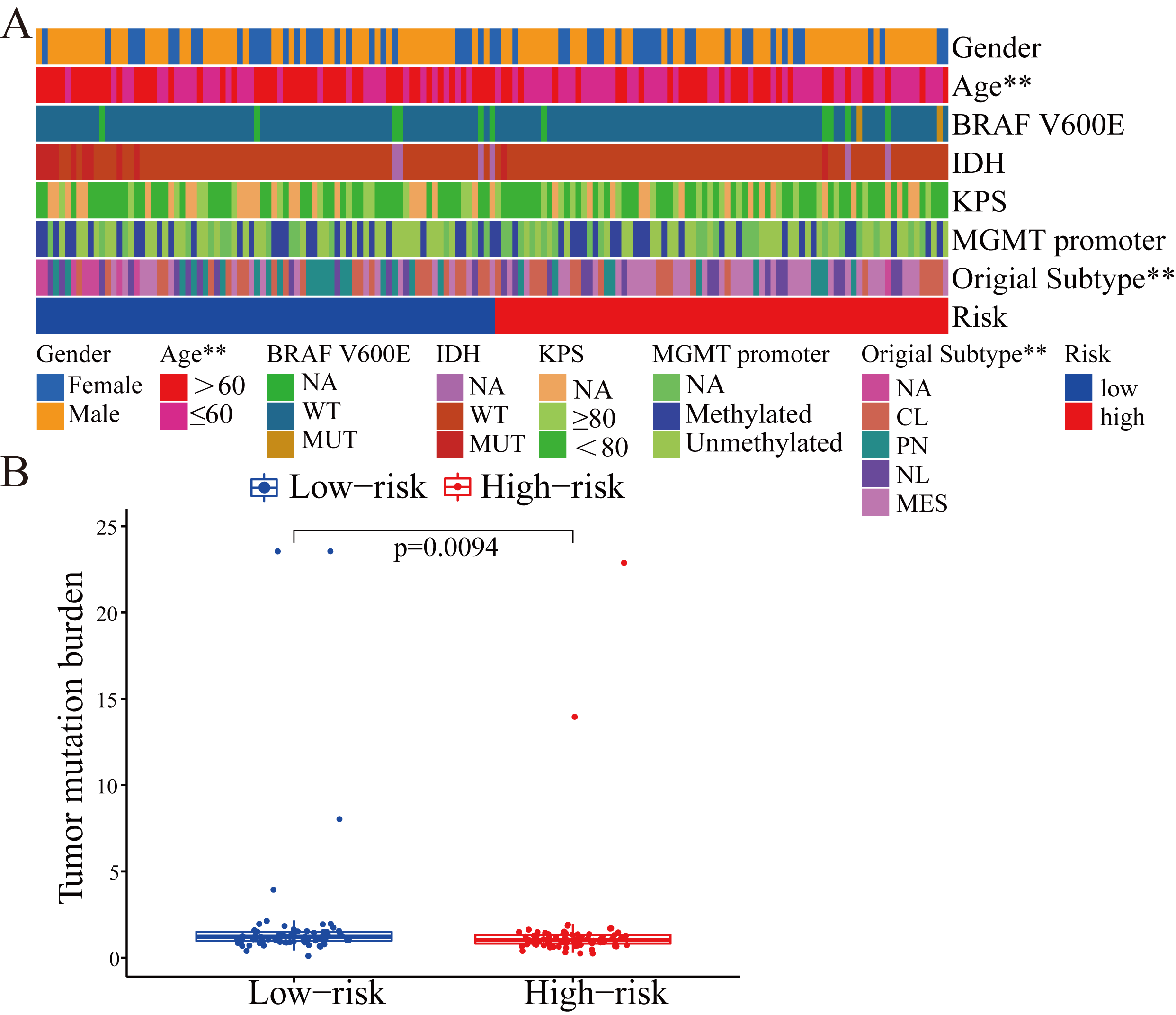

Supplement: Supplementary file 1 [file Image3.TIF]

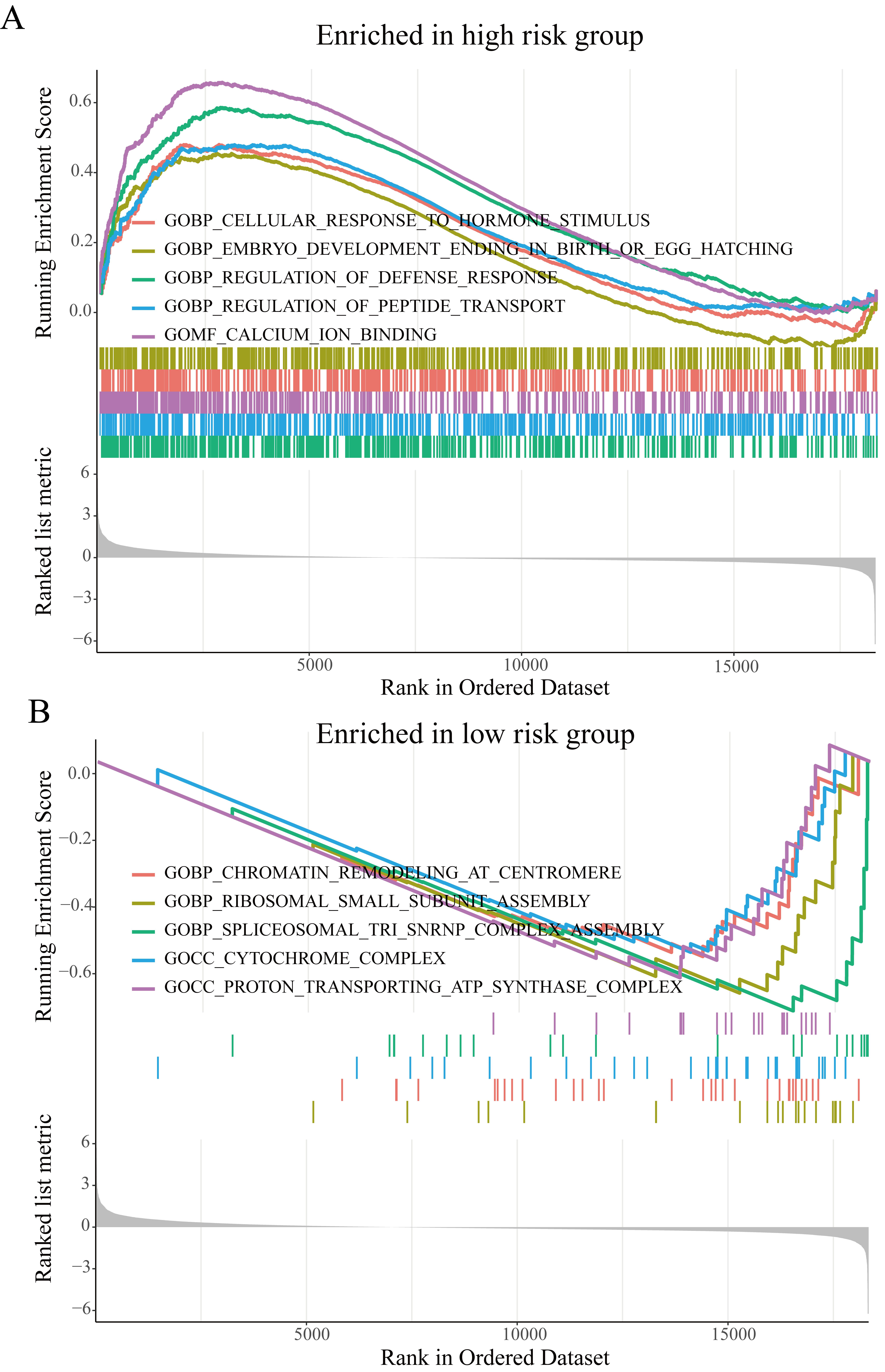

Supplement: Supplementary file 2 [file Image4.TIF]

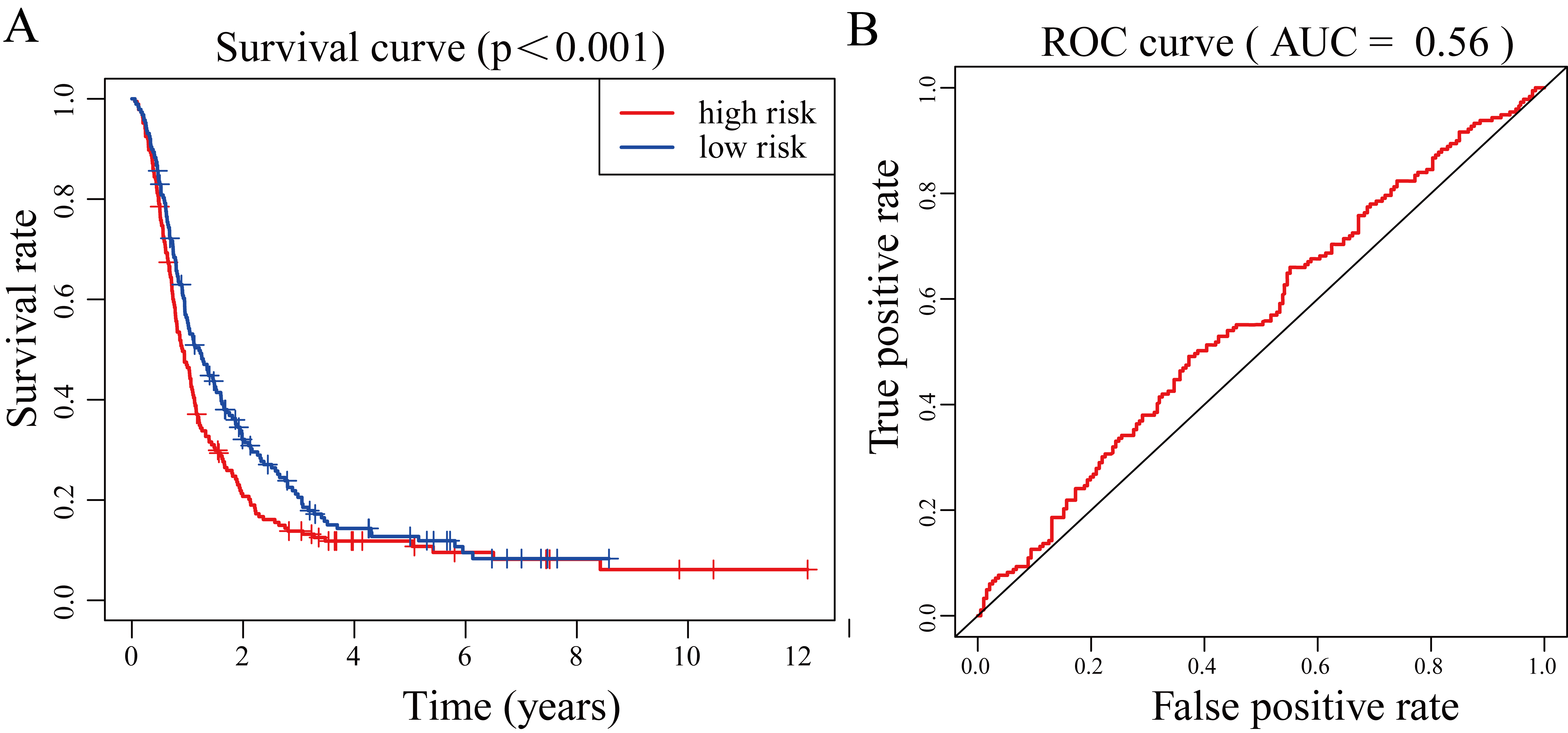

Supplement: Supplementary file 3 [file Image2.TIF]

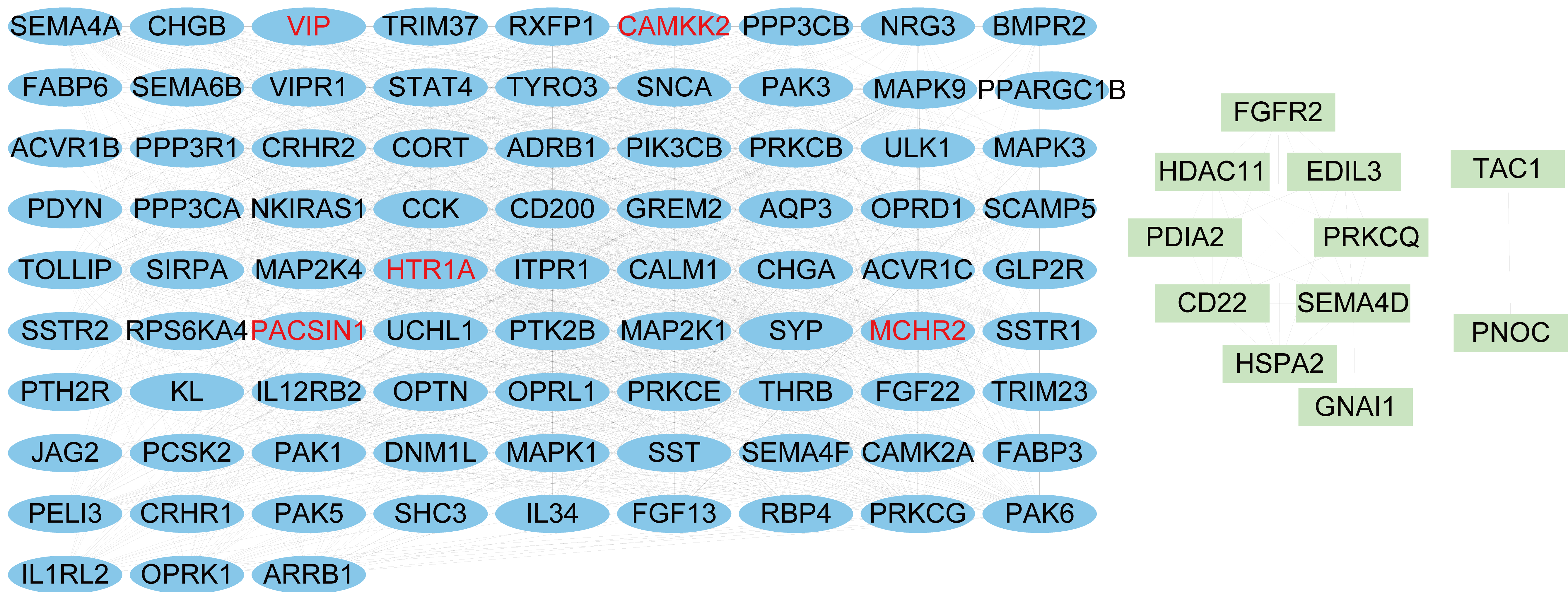

Supplement: Supplementary file 4 [file Image1.TIF]
